# Supplementary material for: The mitochondrial acyl carrier protein (ACP) coordinates mitochondrial fatty acid synthesis with iron sulfur cluster biogenesis
Source: eLife. 2016 Aug 19;5:e17828. doi: 10.7554/eLife.17828 (PMC4991935; doi:10.7554/eLife.17828)
Supplement: Supplementary file 1. — This table describes the name, genotype, and source of all yeast strains used in this investigation. DOI: http://dx.doi.org/10.7554/eLife.17828.017 [file elife-17828-supp1.docx]

| Strain | Genotype | Source |
| --- | --- | --- |
| WT (BY4741) | *MAT*a*, his3 leu2 met15 ura3* | Open Biosystems |
| *acp1*Δ + Acp1-HA | *MAT*a*, his3 leu2 met15 ura3*  *acp1::hygMX ACP1-HA (plasmid)* | This study |
| *ppt2*Δ | *MAT*a*, his3 leu2 met15 ura3*  *ppt2::natMX* | This study |
| WT (W303) | *MAT*a*, his3 leu2 met15 trp1 ura3* | This Study |
| *acp1*Δ + Acp1-V5 | *MAT*a*, his3 leu2 met15 trp1 ura3*  *acp1::hygMX ACP1-V5 (plasmid)* | This Study |
| WT (R1158; BY4741 derivative) | *MAT*a, URA3::CMV-tTA, *his3 leu2 met15* | GE Dharmacon |
| TetO_7_-*ACP1* | *MAT*a, URA3::CMV-tTA, *his3 leu2 met15*  *acp1::Kan_R_TetO_7_-CYC1TATA-ACP1* | This study |
| TetO_7_-*NFS1* | *MAT*a, URA3::CMV-tTA, *his3 leu2 met15*  *nfs1::Kan_R_TetO_7_-CYC1TATA-NFS1* | GE Dharmacon |
| TetO_7_-*ISD11* | *MAT*a, URA3::CMV-tTA, *his3 leu2 met15*  *isd11::Kan_R_TetO_7_-CYC1TATA-ISD11* | This study |
| WT *NFS1*-V5 | *MAT*a, URA3::CMV-tTA, *his3 leu2 met15*  *nfs1::NFS1-V5-natMX* | This study |
| TetO_7_-*ACP1 NFS1-V5* | *MAT*a, URA3::CMV-tTA, *his3 leu2 met15*  *acp1::Kan_R_TetO_7_-CYC1TATA-ACP1*  *nfs1::NFS1-V5-natMX* | This study |
| WT ISD11-V5 | *MATa*, URA3::CMV-tTA, *his3 leu2 met15*  *isd11::ISD11-V5-natMX* | This study |
| TetO_7_-*ACP1 ISD11-V5* | *MAT*a, URA3::CMV-tTA, *his3 leu2 met15*  *acp1::Kan_R_TetO_7_-CYC1TATA-ACP1*  *isd11::ISD11-V5-natMX* | This study |
| WT (DY150) | *MAT*a *ade6 his3-11 leu2-3,112 trp1-1 ura3-52 can1-100(oc*) | This study |
| Gal-*ACP1* | *MATa* *ade6 his3-11 leu2-3,112 trp1-1 ura3-52 can1-100(oc*) *KanMX*::pGal-*ACP1* | This study |
| Gal*-NFS1* | *MAT*a *ura3-52 lys2-801_amber ade2-101_ochre trp1-*Δ*63 his3-*Δ*200 leu2-*Δ*1* cyh2 *nfs1::HIS3* *URA3*::pGal-*NFS1* | Andrew Dancis |
| Gal*-ISD11* | *MAT*a *ura3-52 lys2-801_amber ade2-101_ochre trp1-*Δ*63 his3-*Δ*200 leu2-*Δ*1* cyh2 *HIS3MX6*::pGal-*ISD11* | Andrew Dancis |
| Gal-*ISU1* | *MAT*a *ura3-52 lys2-801_amber ade2-101_ochre trp1-*Δ*63 his3-*Δ*200 leu2-*Δ*1* pRS406-gamma *isu2* (*URA3*) *HIS3MX6*::pGal-*ISU1* | Andrew Dancis |
| Met-*YFH1* | *MAT*a *ade2-1 his3-11 leu2-3,112 trp1-1 ura3-52 can1-100(oc*) *yfh1::HIS3*  pTF63-Met3-*YFH1* (*URA3*) | Jerry Kaplan |
